# Assessment of Intramuscular Verapamil as Pharmacological Countermeasure in a Rat Model of Organophosphate DFP-induced Status Epilepticus

**DOI:** 10.1007/s12640-025-00765-z

**Published:** 2025-10-18

**Authors:** Yam Nath Paudel, Robert E. Blair, Elisa Hawkins, Matthew S. Halquist, Melissa Morgan, Jason Funderburk, Daniel Calvano, Jennifer Koblinski, Hope Richard, Laxmikant S. Deshpande

**Affiliations:** 1 Department of Neurology, School of Medicine, Virginia Commonwealth University, 980599, Richmond, VA 23298 USA; 2 Bioanalytical Shared Resource Laboratory, Departments of Pharmaceutics, Virginia Commonwealth University, Richmond, VA 23298 USA; 3 Department of Pathology, School of Medicine, Virginia Commonwealth University, Richmond, VA 23298 USA; 4 Tissue and Data Acquisition and Analysis Core, VCU Massey Comprehensive Cancer Center, Richmond, VA 23298 USA; 5 Department of Pharmacology and Toxicology, School of Medicine, Virginia Commonwealth University, Richmond, VA 23298 USA

**Keywords:** Organophosphate, Status epilepticus, Verapamil, Neuroprotection, Intramuscular, Pharmacokinetics

## Abstract

Lethal organophosphate (OP) exposure leads to status epilepticus (SE), which, despite standard-of-care (SOC) therapy, is associated with acute mortality and long-term morbidities. Neuronal injury and inflammation are reported following OP-SE, and drugs targeted at these processes have produced beneficial outcomes. Verapamil (VPM) is a calcium-channel blocker used as an antihypertensive drug and has been shown to exhibit neuroprotective and anti-inflammatory actions in experimental models of CNS injuries. Here, we investigated the feasibility of an adjunctive intramuscular (i.m.) VPM therapy in OP Diisopropyl Fluorophosphate (DFP)-induced SE. We also investigated the safety and toxicity of i.m. VPM and compared its pharmacokinetic (PK) profile to oral (p.o.) administration. Rats were injected with DFP (4 mg/kg, s.c.). One minute later, SOC treatment consisting of atropine (0.5 mg/kg, i.m.) and pralidoxime chloride (2-PAM; 25 mg/kg, i.m.) were administered, and at 1-hour post-SE, midazolam (1.78 mg/kg, i.m.) was given. Rats that met the behavioral SE severity criteria (Racine 4–5) were randomized into two treatment groups: those receiving saline (SAL) or VPM (10 mg/kg, i.m. *bid*, 3 days). Histological analysis was conducted to assess neuronal injury and injection-site pathology. In a separate group of rats, PK studies were conducted on blood and brain homogenates treated once with saline or VPM (10 mg/kg, p.o. or i.m.). Our data demonstrated that following DFP-SE, i.m. VPM achieved higher blood and brain levels and exhibited a favorable PK profile compared to p.o. route. VPM therapy did not cause significant muscle pathology and produced a robust neuroprotective response. Neuroinflammatory markers and long-term behavioral outcomes were not included in this study. Our studies provide evidence that the i.m. route is an effective method for delivering VPM following SE, producing significant neuroprotective outcomes compared to treatment with the standard-of-care alone in OP-SE.

## Introduction

Status Epilepticus (SE) is a clinical emergency associated with high mortality and significant neurological morbidities (Betjemann and Lowenstein 2015; Helmstaedter 2007; Kamppi et al. 2024). There are several causes for SE, including stroke, traumatic brain injury, withdrawal from anti-seizure medications, alcohol withdrawal, and exposure to certain toxic chemicals (Trinka et al. 2012). One such category of chemicals that could lead to the rapid induction of SE is the organophosphate (OP) compounds (Deshpande et al. 2010, 2014). These compounds are diverse in their applicability and include commonly used pesticides, industrial chemicals, and additives to jet fuels (Costa 2018). OP compounds also include lethal chemical warfare nerve agents (Bajgar 2004). OP exposure can thus occur accidentally, occupationally, domestically, or during war/terrorism-related scenarios (Anger et al. 2020; Jaga and Dharmani 2003; Sugiyama et al. 2020; White et al. 2016). Furthermore, intentional use of OP compounds for self-harm is also unfortunately common in developing nations (Ajdacic-Gross et al. 2008; Mohanraj et al. 2014).

OP compounds are inhibitors of the enzyme acetylcholinesterase (AChE), leading to accumulation of acetylcholine at the synapse (Aroniadou-Anderjaska et al. 2023; Bajgar 2004; Tsai and Lein 2021). Depending on the OP dose and the level of AChE inhibition, symptoms of OP exposure range from salivation, lacrimation, and headaches to tremors, muscle fasciculations, and seizures. These symptoms could further evolve into SE, respiratory difficulties, and eventually death if prompt medical care is not provided (Chuang et al. 2019; Tattersall 2009). Emergency treatment for OP intoxication includes respiratory support and treatment with a three-drug regimen standard of care (SOC) recommended by the Food and Drug Administration (FDA) to manage hypercholinergic signs. These drugs include atropine (a muscarinic receptor antagonist), pralidoxime chloride (2-PAM; an AChE reactivator), and midazolam (a benzodiazepine to manage seizures) (Chemical Hazards Emergency Medical Management 2013; Eddleston et al. 2008). These emergency procedures have significantly reduced the acute mortality associated with OP exposures. However, despite these treatments, survival from OP exposure is associated with chronic neurological co-morbidities, including memory impairments, mood disorders, and even spontaneous recurrent seizures. Thus, effective treatments are needed to control not only the acute mortality but also lower the incidences of chronic morbidity associated with OP exposures (Jett and Laney 2021; Jett and Spriggs 2020).

Cellular injury and neurodegeneration are key processes underlying the neurological consequences of OP toxicity (Flannery et al. 2016; Naughton and Terry 2018; Tsai and Lein 2021; Voorhees et al. 2016). Our lab has studied neuronal calcium homeostatic mechanisms in rat models of OP toxicity (Deshpande and DeLorenzo 2020). We reported protracted elevations in hippocampal calcium levels using the paraoxon (Deshpande et al. 2014) and diisopropyl fluorophosphate (DFP) (Deshpande et al. 2010) models of OP-induced SE. Calcium-induced calcium release mechanisms were found to mediate these chronic elevations in calcium. Indeed, treatment with ryanodine receptor and inositol-tris-phosphate antagonists such as dantrolene and levetiracetam not only lowered the OP-SE-induced neuronal calcium levels but also produced a neuroprotective effect (Deshpande et al. 2016). Ketamine, which prevents calcium entry by blocking the N-methyl-D-aspartate receptors, has also been neuroprotective in some OP-SE models (Lazar et al. 2024; Lewine et al. 2018). Neuroinflammation has also been reported in OP-SE, and pharmacological treatment aimed at suppressing neuroinflammatory processes is being investigated to improve neurological outcomes following OP intoxication (Gage et al. 2021; Pearson-Smith and Patel 2020; Ramakrishnan et al. 2025; Rojas et al. 2015; Vasanthi et al. 2025; Shah et al. 2024).

Verapamil (VPM) is a phenylalkylamine calcium channel blocker approved by the FDA in the 1980 s for the management of high blood pressure as well as for treating angina and cardiac arrhythmias (Kangilaski and McBride 1982). The non-FDA-approved indications for VPM include its use for treating cluster headaches (Weaver-Agostoni 2013), acute coronary syndrome (Amsterdam et al. 2014), and cerebral vasospasm (Jun et al. 2010). The drug is typically administered orally, and an intravenous formulation is available. The usual dose range is 120 to 360 mg/day administered orally and up to 10 mg parenterally (Whelton et al. 2018). VPM has a long human safety record with minimal side effects and drug-drug interactions (Fahie and Cassagnol 2022). Recent findings regarding the molecular actions of VPM, in addition to calcium-channel inhibition, have demonstrated potent neuroprotective action (Maniskas et al. 2016; Jackson et al. 2018; Jangholi et al. 2020; Zhang et al. 2019), raising the possibility that VPM could also be an effective OP countermeasure drug.

In this study, using a rat model of DFP-induced SE (Deshpande et al. 2010), we compared the pharmacokinetics of VPM when administered orally (p.o.) or intramuscularly (i.m.). The i.m. route was selected given the necessity for ease of administration during an OP-based mass casualty scenario. VPM is also a substrate for P-glycoprotein (P-gp), a transmembrane protein that moves drugs out of the cell (Ledwitch et al. 2016; Summers et al. 2004). Therefore, we also measured brain levels under both SE and no-SE conditions to investigate the brain penetrance and availability of VPM. We also investigated the neuroprotective efficacy of adjunctive i.m. VPM therapy in various brain regions following DFP-induced SE when co-administered with the standard FDA-approved three-drug regimen of atropine, 2-PAM, and midazolam indicated for OP intoxication. Muscle pathology at the injection site was also evaluated to assess the safety of repeatedly administering i.m. injections. Finally, a stability analysis of our VPM formulation was also conducted.

## Materials and methods

### Drugs and Chemicals

DFP (purity > 90%) was obtained from Chem Service Inc. (West Chester, PA). Verapamil hydrochloride, atropine sulfate, and 2-PAM were obtained from Millipore-Sigma (St. Louis, MO). Midazolam (5 mg/mL) was obtained from Med-Vet International (Mettawa, IL). To prepare a working DFP solution (4 mg/mL), the desired quantity of DFP was removed using a Hamilton syringe and added to a glass bottle containing ice-cold phosphate-buffered saline (PBS). The solution was then gently vortexed. The bottle was kept on ice, and syringes were drawn and kept on ice until the time of subcutaneous (s.c.) injections. Time on ice (between dilution in PBS and injection) was never more than ten minutes. VPM, atropine, and 2-PAM were dissolved in saline (SAL, 0.9% NaCl) and then sterile-filtered. All the drugs were prepared fresh on the day of the experiment.

### Animals

All animal use procedures were conducted per the National Institutes of Health Guide for the Care and Use of Laboratory Animals and were approved by Virginia Commonwealth University’s Institutional Animal Care and Use Committee. Male and Female Sprague-Dawley rats were obtained from Envigo (Indianapolis, IN) at 9 weeks of age. Animals were housed two per cage at 20–22 °C with a 12-hour light: 12-hour dark cycle (lights on from 6:00 to 18:00 h) and had free access to food and water.

An equal number of male and female rats were randomized into groups consisting of the two experimental conditions (SAL-control or DFP-SE), the two treatment conditions (SAL or VPM), and for the two routes of administration (p.o. and i.m.). A total of 136 rats were used in this study as follows- PK profiling: 24 rats (*n* = 3/condition), VPM brain level estimations: 16 rats (*n* = 4/condition), Muscle pathology assessments: 32 rats (*n* = 4/condition), and Neuroprotection studies: 64 rats (*n* = 8/condition).

### Induction of DFP-SE

**Fig. 1:**
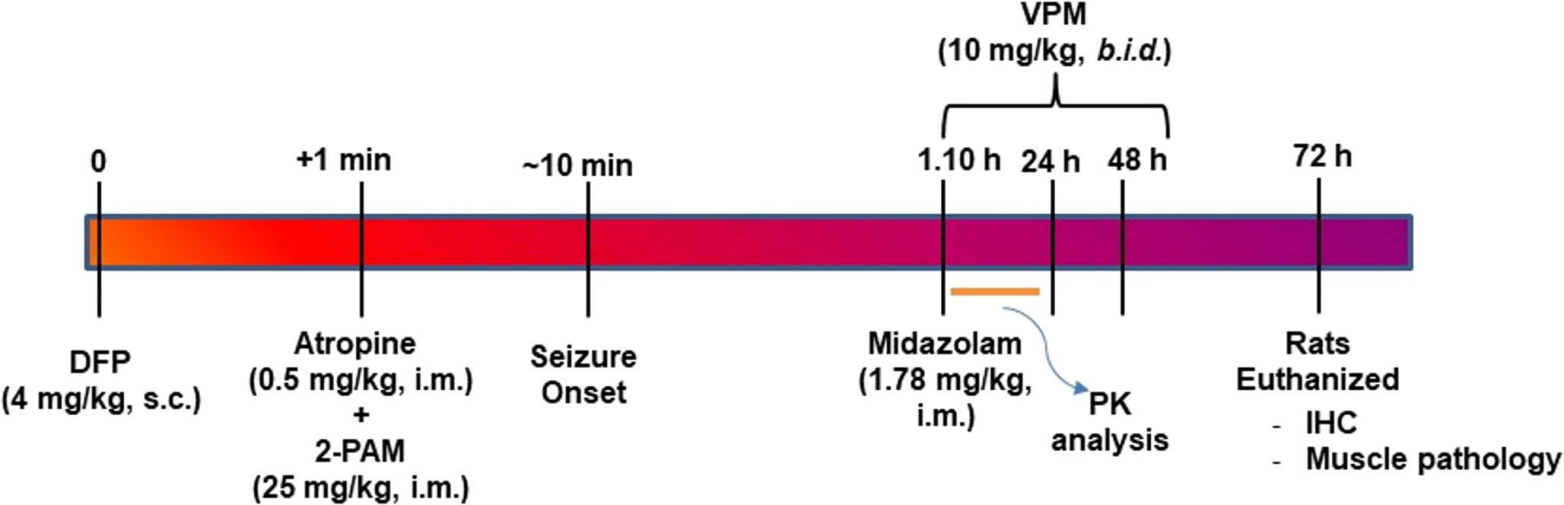
Timeline for induction of DFP-SE and VPM interventions post SE At time 0, Sprague-Dawley rats (both male and female) were injected with the OP agent DFP (4 mg/kg, s.c.). One minute later, atropine sulfate (AtSO_4_; 0.5 mg/kg, i.m.) and 2-PAM (25 mg/kg, i.m.) were injected. SE onset was approximately 10 min. One hour following DFP-SE onset, rats were administered midazolam (1.78 mg/kg, i.m.) was injected. For PK studies, VPM (10 mg/kg) was administered once via the p.o. or i.m. route, and blood and brain samples were collected at various time points up to 24 h. For histological studies, rats were injected with saline or VPM (10 mg/kg, i.m., *b.i.d.*) for three consecutive days alternating between right and left quadriceps. Mortality was assessed daily till the fourth day, when the rats were euthanized and perfused for brain and quadricep muscle collection

As shown in Fig. 1, in accordance with our previously published protocol for inducing DFP-induced SE (Blair et al. 2024; Deshpande et al. 2010), separate cohorts of rats were injected with DFP (4 mg/kg, s.c.) or SAL (0.1 ml/kg). One minute later, atropine (0.5 mg/kg, i.m.) and 2-PAM (25 mg/kg, i.m.) were injected to permit survival and progression into SE. DFP exposure produced signs of progressive cholinergic hyperstimulation culminating in continuous seizures (SE). We utilized the Racine criteria for assessing SE severity (Racine 1972a, b). The criteria for the behavioral seizure score were: 0- behavioral arrest, hair raising, excitement, and rapid respiration; 1- mouth movement of lips/tongue, vibrissae movement, and salivation; 2- head “bobbing”/clonus; 3- forelimb clonus; 4- clonic rearing, and 5- clonic rearing with loss of postural control. A Racine score of 4–5 was used as the criterion to mark the onset of SE, approximately 5–10 min after the DFP injection. Our previous studies have shown that this Racine score correlates with the tonic-clonic phase of SE and is accompanied by an EEG frequency of > 6 Hz, which meets the clinical criteria of SE (Blair et al. 2024; Deshpande et al. 2010). At 1-h post-SE onset, midazolam (1.78 mg/kg, i.m.) was administered to suppress SE. Midazolam administration was delayed by 1 h post-DFP-SE onset to simulate a practical therapeutic window for first responders or hospital admission following mass casualty events (Deshpande et al. 2010; Guignet et al. 2020; Reddy et al. 2021). These doses were determined using human equivalents calculated using a human-to-rat dose translation equation (Nair and Jacob 2016; Reagan-Shaw et al. 2008). Age-matched control rats received either SAL or VPM with the exclusion of DFP.

### VPM Treatment

Rats that met the SE severity criteria (Racine 4–5) and received midazolam at 1-h post-DFP-SE onset were randomized into two treatment groups: those receiving saline (SAL) or VPM. Previous studies indicated that VPM could be administered i.p. in rodent seizure models at doses up to 40 mg/kg (George et al. 2000; Vezzani et al. 1988; Wurpel and Iyer 1994). A pilot study with two doses of VPM (10 and 20 mg/kg, i.m.) at 1 h after DFP-SE was conducted in rats (*n* = 5). Rats safely tolerated both doses in these pilot studies. For this study, we chose a 10 mg/kg twice-daily treatment regimen as this dose was closer to the human-equivalent dose (Reagan-Shaw et al. 2008) and would allow for a stable plasma level given a T_1/2_ of 5 h in humans (Follath et al. 1986; McAllister and Kirsten 1982). Thus, VPM was administered concomitantly with the first dose of midazolam at 60 min post-DFP-SE onset and subsequently twice daily for up to 72 h, for a total of six VPM doses. The control group was given i.m. SAL injections for the same duration and frequency. For i.m. administration, VPM or SAL was administered in the quadriceps muscle using a 28-gauge needle. The injection volume was 0.1mL/kg. Injection sites were alternated between the lateral and contralateral sides for the twice-daily dosing protocol. Manifestations of distress and discomfort (such as limping, licking the injection site, and other ambulation-related issues) were subjectively graded as: none, mild, moderate, and severe. Mild discomfort was noted on the last day of i.m. injections, which quickly resolved without any intervention. No ambulatory deficits were observed in these rats. Muscle pathology was conducted as described below.

### Pharmacokinetic Analyses

For the PK study, rats were dosed once with SAL or VPM (10 mg/kg). Blood samples were obtained in duplicate from 3 to 4 rats at each of the 10 time points for the two routes of administration (i.m. or p.o.) following administration of either saline or DFP. Following anesthesia, rats were euthanized and blood samples were collected through cardiac ventricles at 0, 5 min, 30 min, and at 1, 2, 4, 6, 12, 18, and 24 h following VPM administration. The blood was placed in an EDTA microcuvette and frozen at −80 °C until analyzed. Concurrently with blood collection, the brain was rapidly dissected, and the cortex was removed and snap frozen in isopentane (−15 °C) and stored at −80 °C until use. VPM levels were measured in whole blood and cortex using liquid chromatography tandem mass spectrometry (LC-MS/MS). The linear range was 0.05–1200 ng/mL for VPM.

### Sample Preparation

50 µL of rat whole blood was analyzed for VPM levels using a protein precipitation extraction method. Briefly, each aliquot was transferred to a 1.5 mL micro-centrifuge tube, and 500 µL of extraction solvent consisting of 70% methanol and 30% water was added, following the 50 µL of internal standard solution (AT-d 7 and LV-d 6 at a concentration of 500 ng/mL). Sample tubes were vortexed for 1 min and sonicated for 30 min. Samples were then centrifuged for 10 min at 13.2 g and transferred to an amber vial. VPM was analyzed using LC-MS/MS with multiple reaction monitoring (MRM) transitions on an AB Sciex 6500 + Qtrap (Sciex, USA) with an LC-20-AD (Shimadzu, USA) UPLC system. Chromatographic separation was performed on a Zorbax Eclipse Plus C18 Column (2.1 × 100 mm, I.D. 3.5 μm, Agilent Technologies, USA). The column temperature was ambient; the flow rate was 1.0 mL/min, and the injection volume was 1 µL. The mobile phase A consisted of 0.2% formic acid in water, and mobile phase B in methanol. Isocratic conditions were used at acetonitrile (95%) and 0.2% formic acid (5%) in reagent water. Data were analyzed with Analyst 1.6 Quantitation Wizard in accordance with the Bioanalytical Laboratories Standard Operating Procedures.

### Perfusion and Tissue Collection

At 96 h post-DFP-SE, a separate group of rats underwent perfusion and fixation procedures to obtain brain and muscle tissue. Following induction of deep anesthesia with ketamine/xylazine (75 mg/7.5 mg/kg, i.p.), rats underwent transcardial perfusion with isotonic saline. Then, they were perfused with 250 mL of 4% paraformaldehyde in 0.1 M phosphate buffer (pH 7.4). Brains and quadricep muscles were removed and allowed further fixation for 24 h in buffered 4% paraformaldehyde at 4 °C. Brains were then cryoprotected in 30% sucrose in 0.1 M phosphate buffer (pH 7.4) for 3 days at 4 °C. Brains were then embedded in OCT by snap freezing in isopentane (−15^o^ C) and stored at −80 °C until use. Post-fixed muscle was transferred to a 70% ethanol solution and processed as described below.

### Fluoro-Jade C Staining

Coronal brain sections were prepared using a Leica CM3050S cryostat (Leica Biosystems, Deer Park, IL) and adhered to glass slides (Superfrost Plus; Fisher Scientific, Pittsburgh, PA) and stored at −80 °C until use. Briefly, 20 μm-thick sections were collected between (−1.8 mm to −4.56 mm Bregma, based on rat anatomic atlas (Paxinos and Watson 2006; Paxinos et al. 1980). The sectioning spans the entire dorsal hippocampal formation and includes the somatosensory cortex, thalamus, amygdala, and piriform cortex. Each slide contained sections representing all experimental groups (age-matched control, DFP-SAL, DFP-VPM). Every 20th section through the rat hippocampus is selected from at least six animals for labeling and staining (Deshpande et al. 2016; Guignet et al. 2020; Reddy et al. 2021; Rojas et al. 2021, 2022; Wu et al. 2018).

Slides were dried in a desiccant chamber at 55 °C for 30 min prior to staining. Slides were first incubated in a solution of 1% NaOH in 80% ethanol for 5 min, followed by hydration in 70% ethanol and then in ddH2O for 2 min each. Slides were then incubated in a 0.06% KMnO_4_ solution for 10 min, followed by washing in ddH_2_O for 2 min. Slides were then stained in a 0.0004% Fluoro-Jade C (FJC) solution in 0.1% acetic acid for 20 min. Stained slides underwent three washes in ddH2O for 2 min each and were then dried in a desiccant chamber at 55 °C for 30 min. Stained slides were then cleared with xylene for 5 min and cover-slipped with DPX mounting agent. FJC-stained sections were viewed under 20X magnification on an Olympus IX-70 microscope under brightfield or fluorescein/FITC filter settings, and 16-bit grayscale digital images were acquired with a digital CCD camera (ORCA-ER; Hamamatsu Corp) using MetaMorph7.8 (Molecular Devices, San Jose, CA). All microscope and camera settings for FJC image acquisition were constant throughout. Grayscale images were analyzed using ImageJ 1.53 (NIH). Specific FJC-stained cells were measured using auto-threshold segmentation (Yen method) of selected regions of interest and counts of stained cells within threshold were detected using the analyze particle module with a size range of 50–150 pixels and cell counts/mm^2^ were calculated (Deshpande et al. 2016; Rojas et al. 2021, 2022).

### Assessment of Muscle Pathology

To evaluate the safety of i.m. VPM administrations, quadriceps muscle specimens were obtained at 1 day and 3 months post the last i.m. injection of the VPM treatment regimen. To conserve animal numbers, muscle specimens for acute toxicity assessment were obtained from the same rats used for dissecting the brain for FJC staining. A separate cohort of rats was utilized for the 3-month time point. These time points were selected to assess whether repeated VPM injections caused any muscle injury at the site of injection, either acutely or chronically. Muscle specimens were obtained as described in the perfusion section above. Muscle samples were embedded in paraffin, and 5-micron-thick sections were obtained. These sections were then stained with Hematoxylin and Eosin (H&E) for histopathological analysis. Automated H&E staining was performed with the Dako CoverStainer (Agilent). A standard four-point inflammation scale was used for scoring (Deshpande et al. 2020; Patterson et al. 2008) as described in Table 1. The score for each site was the average of the scores for each of the three tissue blocks.

**Table 1:** Inflammation scale for assessment of injection site muscle pathology

Score	Fibrosis	Infiltrates
0 = Not present	0	None present
1 = Minimal	< 1 mm	Few, widely scattered infiltrates
2 = Mild	1 to 1.9 mm	Scattered infiltrates, minimal small clusters
3 = Moderate	2 to 3.9 mm	loosely packed fields, many small clusters
4 = Marked	> 4 mm	Dense infiltrates, often packed fields

### Stability Analysis of VPM Formulation

VPM formulation (10 mg/mL in sterile saline) in multiple glass bottles was stored at 25 °C ± 2 °C and 60 ± 5% Relative Humidity. At the respective time points (Day 0, 90, 203), three bottles were randomly chosen, and the stability of the VPM formulation was assessed under ICH QA1 (R2) guidelines [Stability Testing of New Drug Substances and Products] and using a similar USP VPM injection monograph (USP29-NF24).

Similar to the USP29-NF24 VPM monograph, a system suitability solution was prepared at 1.9 mg/mL of USP Verapamil Hydrochloride RS and 1.5 mg/mL of USP Verapamil Related Compound B RS in mobile phase (see Table 2). The VPM standard solution (2.5 mg/mL) was also prepared in the mobile phase. Impurity Standard solution: 2.5 mg/mL of USP Verapamil Hydrochloride RS, and 7.5 µg/mL each of USP Verapamil Related Compound A RS, USP Verapamil Related Compound E RS, and USP Verapamil Related Compound F RS in Mobile phase. Filter the samples and standards using a syringe filter into 1 mL autosampler tubes.

**Table 2:** HPLC conditions for VPM concentration determination

Column	4.6-mm ×15-cm; 5-µm packing L1
Mobile Phase	Acetonitrile, 0.015 N Sodium Acetate, 3.3% acetic acid, and 2-aminoheptane (30:70:0.5)
Needle Wash	60:30:10 Acetonitrile: Methanol: Water
Flow Rate	0.9 mL/min
Injection Volume	10 µL
Detector	UV 278 nm, Range 200–400 nm for Identification
Column Temperature	30 °C
Sample Temperature	6 °C
Run Time	4 min (NLT 4 times retention time of verapamil)
Software	Waters Empower

### Experimental Rigor and Data Analysis

Male and Female rats were housed in separate rooms. All the drugs were prepared fresh daily and belonged to the same respective batch to minimize experimental variability due to possible minor differences between drug lots. DFP-SE induction protocol and VPM treatment were carried out on separate days for male and female rats. Rats were randomized, ignoring sex and the estrous cycle stages. Experimental groups were blinded until the data were analyzed. The study excluded animals that did not present with SE onset or failed to achieve a Racine score of 4 or higher. Two independent observers assessed the severity of SE. Data analysis was performed using GraphPad Prism 10 software. Data normality was tested with the Shapiro-Wilk test. Data represented as mean ± SD.

For PK analysis, model-independent methods were used to estimate the pharmacokinetic parameters of VPM in rat blood samples. The area under the curve (AUC) was determined utilizing the parameters programmed in Microsoft Excel. AUC from time zero to the last sampling time was calculated using the linear trapezoidal rule. The AUC from the last sampling time was extrapolated to infinity by dividing the last measured plasma concentration by the terminal elimination rate constant. Cmax and Tmax were obtained directly from the concentration-time curves. A two-way ANOVA was utilized to evaluate sex and treatment differences and a post-hoc test was employed as described below. A *p* <.05 was considered to indicate statistical significance. PK data and neurodegeneration were analyzed using a two-way ANOVA followed by Sidak’s multiple comparison test. VPM brain levels were assessed with a two-way ANOVA followed by Bonferroni’s multiple comparison test, while muscle inflammation was evaluated using a two-way ANOVA.

## Results

### Pharmacokinetic Studies after i.m. And p.o. Treatment with VPM

VPM blood levels were measured in control rats (no DFP-SE) at various time intervals after administration by i.m. and p.o. routes. Table 3 presents data on the pharmacokinetics (PK) of VPM treatment in control rats. In both control male and female rats, a significant increase in bioavailability (AUC) was observed when VPM was administered i.m. compared to p.o. administration. Similarly, a higher drug concentration in plasma (Cmax) was also noted, however, this effect was not significant. The time to peak VPM level (Tmax) was delayed and a significant reduction in terminal half-life (t_1/2_) was noted following i.m. dosing under control conditions (*n* = 3–4 rats/group. *p <.05, two-way ANOVA, post-hoc Sidak’s multiple comparison test).

**Table 3:** Summary of i.m. To p.o. Pharmacokinetics for verapamil in control rats

Verapamil (males)					
	AUCtrap [ng/ml*min]	AUC∞ [ng/ml*min]	tmax [min]	t1/2 [min]	Cmax [ng/ml]
IM	153,774 ± 27,333*	18,717,055 ± 3,770,476*	60 ± 0	159 ± 6*	994 ± 295
PO	16,591 ± 4557	2,109,810 ± 370,821	46 ± 16	389 ± 35	379 ± 376
Verapamil (females)					
	AUCtrap [ng/ml*min]	AUC∞ [ng/ml*min]	**tmax [min]**	**t1/2 [min]**	**Cmax [ng/ml]**
IM	117,482 ± 12,713*	18,316,706 ± 478,279*	50 ± 17	152 ± 2*	773 ± 118
PO	27,597 ± 3682	6,376,058 ± 987,891	37 ± 12	248 ± 54	213 ± 59

Verapamil (10 mg/kg) was administered to control rats separately via intramuscular (i.m.) or oral (p.o.) route. Animals were sacrificed at various time points after injection, and blood was collected for analysis. Descriptive pharmacokinetic parameters are C_max_, maximal plasma concentration, where plasma concentration is in ng/ml; Tmax, time to reach C_max_; t½, terminal half-life; AUC_trap_, area under the plasma concentration time curve 0→∞ via trapezoidal method; AUC∞, area under the plasma concentration time curve extrapolated to infinity (*n* = 3–4 rats/group. *p <.05, two-way ANOVA, post-hoc Sidak’s multiple comparison test)

Next, we investigated the PK profile of i.m. VPM administered after DFP-SE. As shown in Table 4, non-compartmental analysis revealed comparable values for various PK parameters in male and female rats. However, some differences were noted. Compared to the control (no-DFP SE) condition, an even higher Cmax was obtained in male rats after DFP-SE. Similarly, while the AUC for i.m. VPM was higher in DFP-SE, this parameter was significantly higher in female rats. Furthermore, the time to Tmax after i.m. VPM administration in DFP-SE male rats was decreased compared to control males, but not observed in female DFP-SE rats. Finally, a significant reduction in terminal half-life (t_1/2_) was noted following i.m. dosing under SE conditions in male but not female rats (*n* = 3–4 rats/group. *p <.05, two-way ANOVA, post-hoc Sidak’s multiple comparison test).

**Table 4:** Summary of i.m. Pharmacokinetics for verapamil in control and DFP-SE rats

Verapamil (DFP, males)					
	AUCtrap [ng/ml*min]	AUC∞ [ng/ml*min]	tmax [min]	t1/2 [min]	Cmax [ng/ml]
IM-CTL	153,774 ± 27,333	18,717,055 ± 3,770,476	60 ± 0	159 ± 7	994 ± 295
IM- SE	223,027 ± 40,778	33,138,419 ± 1,033,182	46 ± 15	136 ± 7*	1763 ± 300*
Verapamil (DFP, females)					
	AUCtrap [ng/ml*min]	AUC∞ [ng/ml*min]	**tmax [min]**	**t1/2 [min]**	**Cmax [ng/ml]**
IM-CTL	117,482 ± 12,714	18,316,706 ± 478,279	50 ± 17	152 ± 2	773 ± 118
IM- SE	285,769 ± 158,193	110,757,434 ± 97,746,499*	50 ± 17	139 ± 10	708 ± 256

Verapamil (10 mg/kg) was administered to Control (CTL) and DFP (SE) rats via the intramuscular (i.m.) route. Animals were sacrificed at various time points after injection, and blood was collected for analysis. Descriptive pharmacokinetic parameters are C_max_, maximal plasma concentration, where plasma concentration is in ng/ml; Tmax, time to reach C_max_; t½, terminal half-life; AUC_trap_, area under the plasma concentration time curve 0→∞ via trapezoidal method; AUC∞, area under the plasma concentration time curve extrapolated to infinity (*n* = 3–4 rats/group. *p <.05, two-way ANOVA, post-hoc Sidak’s multiple comparison test)

**Fig. 2:**
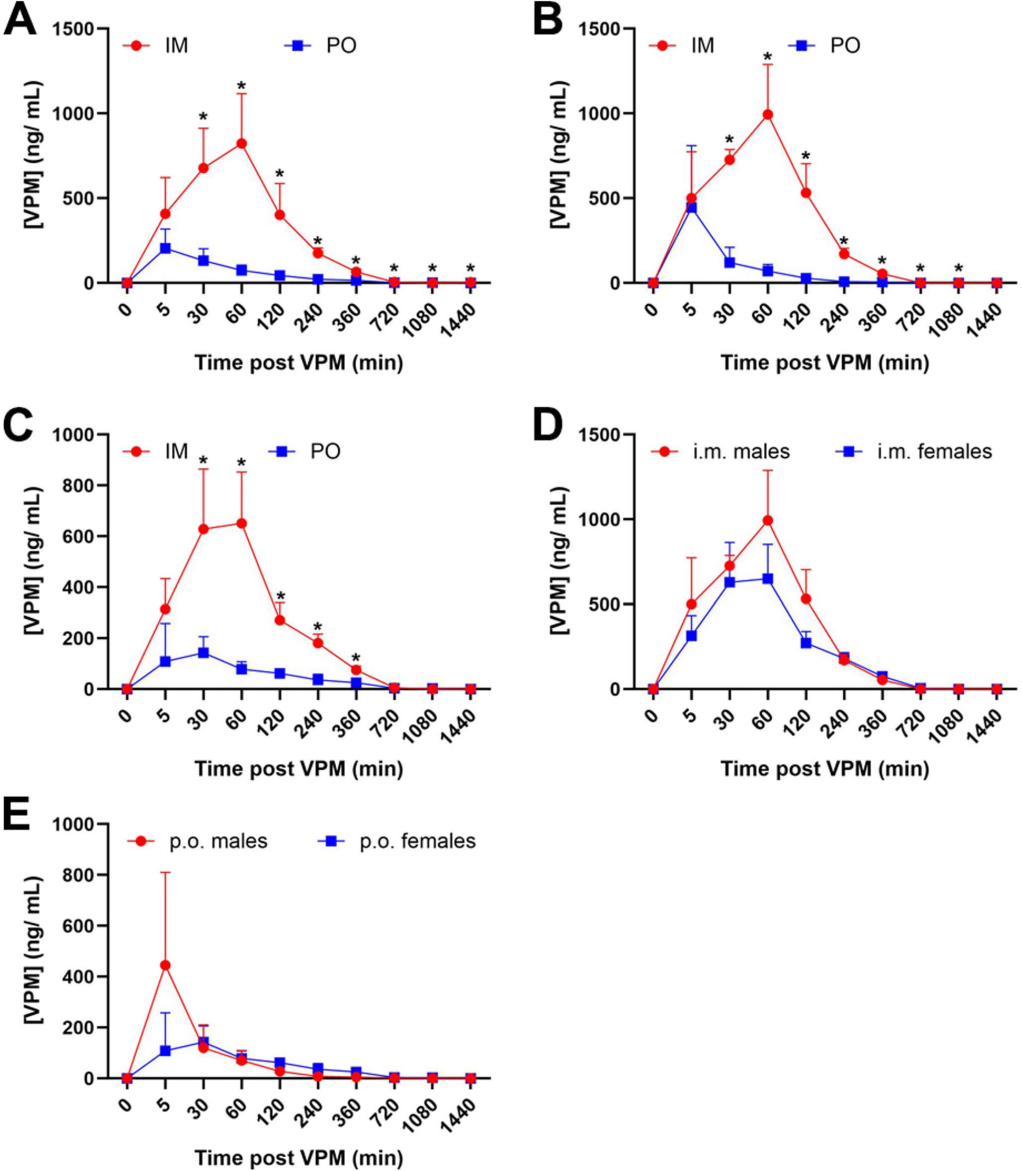
Blood levels of VPM following p.o. and i.m. administration. Comparison of the PK profile of VPM (10 mg/kg) following p.o. and i.m. administration in control rats. Levels of VPM were analyzed using LC-MS/MS in blood specimens collected from control male and female rats at time points ranging from 0 min to 1440 min (24 h). **A.** significantly higher and sustained levels of VPM were noted after i.m. compared to p.o. dosing. **B**,** C.** Significantly higher VPM levels were achieved in both male and female rats. **D**,** E.** No sex differences were noted for VPM levels following i.m. or p.o. administration. (Each data point represents the mean ± SD of concentration values (ng/ml) from *n* = 3 rats. *p <.05, two-way ANOVA, post-hoc Sidak’s multiple comparison test)

Figure 2 shows a comparison of plasma concentration (ng/mL) versus time profiles for VPM in male and female rats for the two dosing routes. The i.m. route provided significantly higher and sustained blood VPM levels in both male and female rats (Fig. 2A). Significantly higher VPM levels were reached within 30 min of i.m. injection and remained significantly elevated at all subsequently measured time points compared to p.o. dosing in both the male (Fig. 2B) and the female (Fig. 2C) rats. No significant differences in VPM levels were noted between the sexes for either i.m. or p.o. administration. routes (Fig. 2D, E) (*n* = 3–4 rats/group. *p <.05, two-way ANOVA, post-hoc Sidak’s multiple comparison test).

### Brain Levels of VPM

**Fig. 3:**
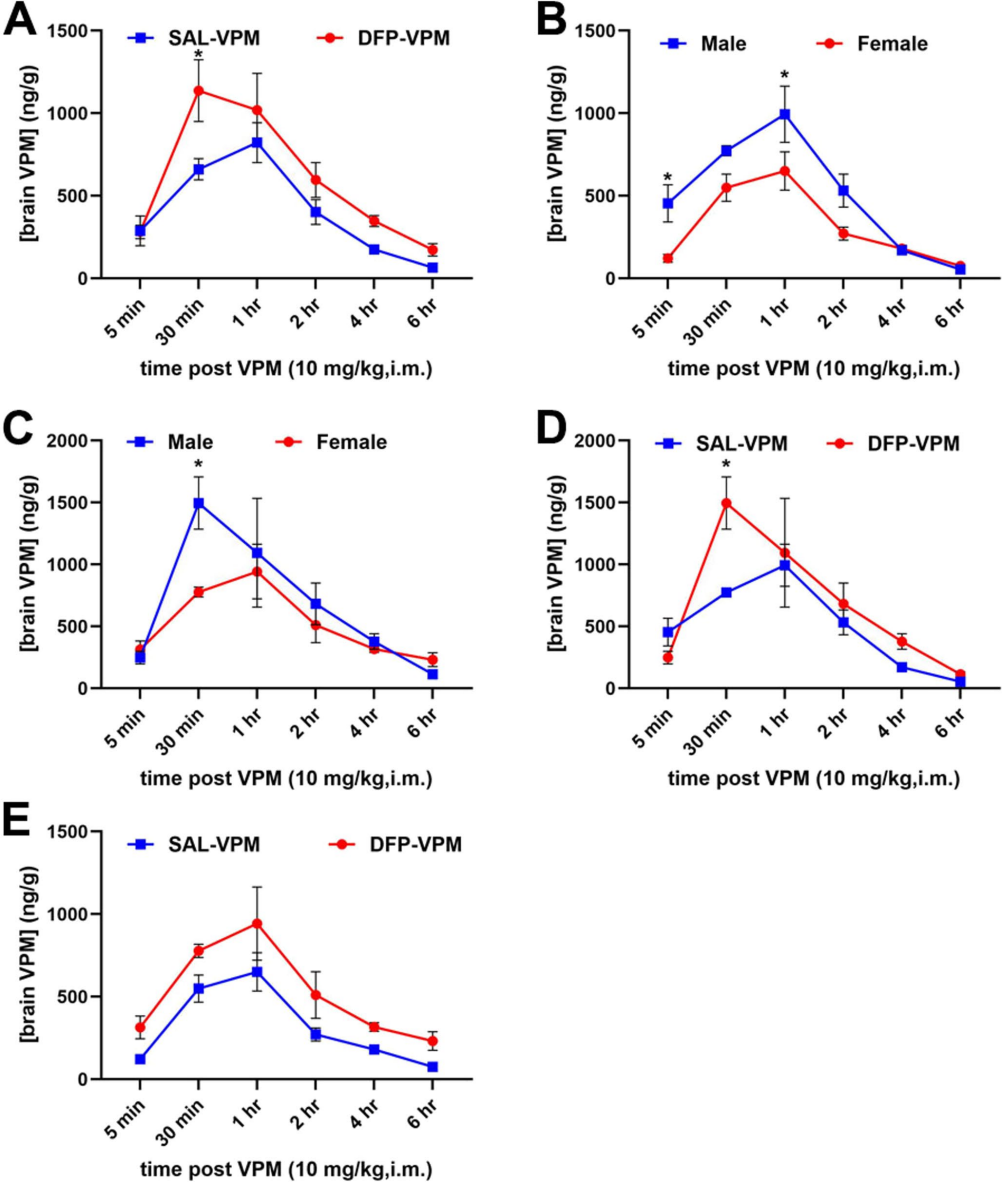
Brain levels of VPM following i.m. administration. VPM (10 mg/kg, i.m.) was administered once to control and DFP-SE male and female rats, and the brain was dissected at various time points ranging from 0 to 24 h. The concentration of cortical VPM was then estimated using LC-MS/MS. **A.** Mixed-sex analysis showed higher levels of brain VPM in both control and DFP-SE conditions. **B-C.** Higher levels of VPM were observed under the DFP-SE condition compared to the control condition in both male (**B**) and female (**C**) rats. **D-E.** Male rats showed higher VPM levels, which peaked earlier than those in female rats under both control (**D**) and DFP-SE conditions (**E**). Significant differences at each time point are indicated (*n* = 3–4 rats/timepoint/group, *p <.05, two-way ANOVA, Bonferroni’s multiple comparison test)

Upon i.m. administration, VPM was detected in the brains of male and female rats under control and DFP-SE conditions (Table 3). Mixed-sex analysis revealed differences in VPM brain levels under the control and DFP-SE conditions. Following DFP-SE, male rats attained significantly higher VPM concentrations as early as 30 min, and this level was sustained for up to 1 h. Subsequently, male rats continued to exhibit higher VPM concentrations for up to 4 h. Beyond this time point, the VPM concentration in male rats began to decline while remaining stable in female rats. Significantly higher VPM levels were observed in female rats at the 6-hour time point. At the 18-hour time point, VPM was undetectable in male rats but measurable in female rats (Fig. 3; *n* = 3–4 rats/timepoint/group, *p <.05, two-way ANOVA, Bonferroni’s multiple comparison test).

### Muscle Safety after i.m. VPM Treatment Protocol

**Fig. 4:**
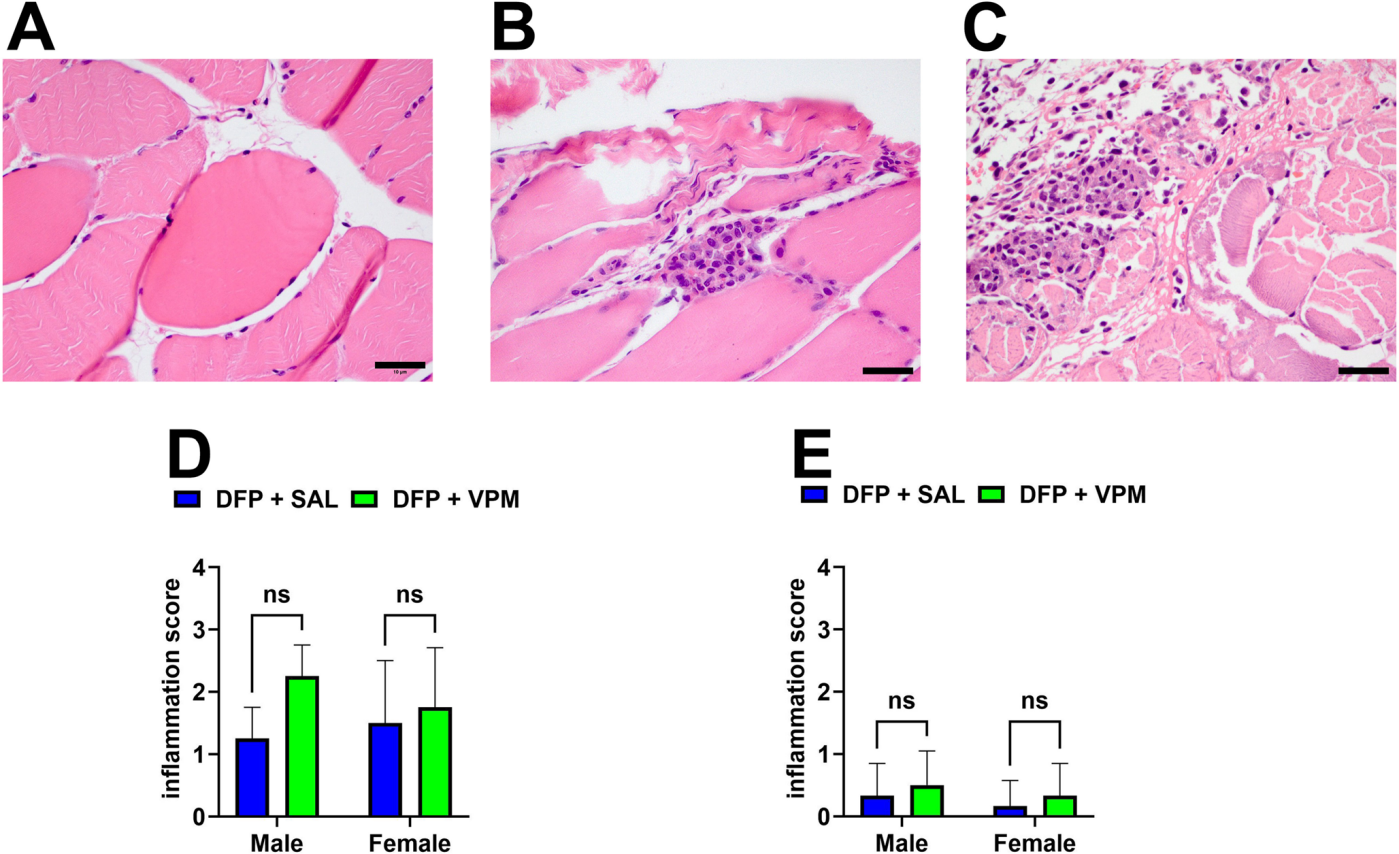
Acute and chronic muscle pathology following i.m. VPM therapy. H&E-stained quadriceps muscles were assessed for the presence and extent of infiltrates and scored on a four-point inflammation scale (Table 1). Representative pictographs depicting the severity of quadricep muscle inflammation commonly noted following i.m. administrations in this study. (**A**) few scattered infiltrates (score = 1). (**B**) few small clusters (score = 2) and (**C**) loosely packed field with many small clusters (score = 3) (black bar scale 10 µM). These pictographs were scored for severity and analyzed. The data represent the muscle damage score for the i.m. VPM therapy protocol (twice-daily for 3 days alternating between right and left quadriceps) when evaluated 1 day and 3 months after the end of the injections regimen in the following groups: DFP + SAL (blue) and DFP + VPM (green) in male and female rats. No significant sex or treatment differences in the inflammation scores were noted **(D)** acutely or (**E**) chronically (data represented as mean inflammation score ± SD; *n* = 4 rats/group, two-way ANOVA, *p <.05)

Inflammation was assessed using a four-point scale (Fig. 4A-C; Table 1). As illustrated in Fig. 4D, one day after the last i.m. injection, the pathological scores for quadricep muscle inflammation were not significantly different between DFP-SE rats treated with SAL and those treated with VPM. As illustrated in Fig. 4E, three months post-injection, the muscle pathology score for the DFP-SE rats treated with SAL control did not differ significantly from that of the VPM tissue. No gender-specific differences were noted in inflammation scores between SAL and VPM treatment at both the acute and chronic time points (*n* = 4 rats/group, two-way ANOVA, *p <.05).

###  Assessment of the Neuroprotective Efficacy of i.m. VPM

**Fig. 5:**
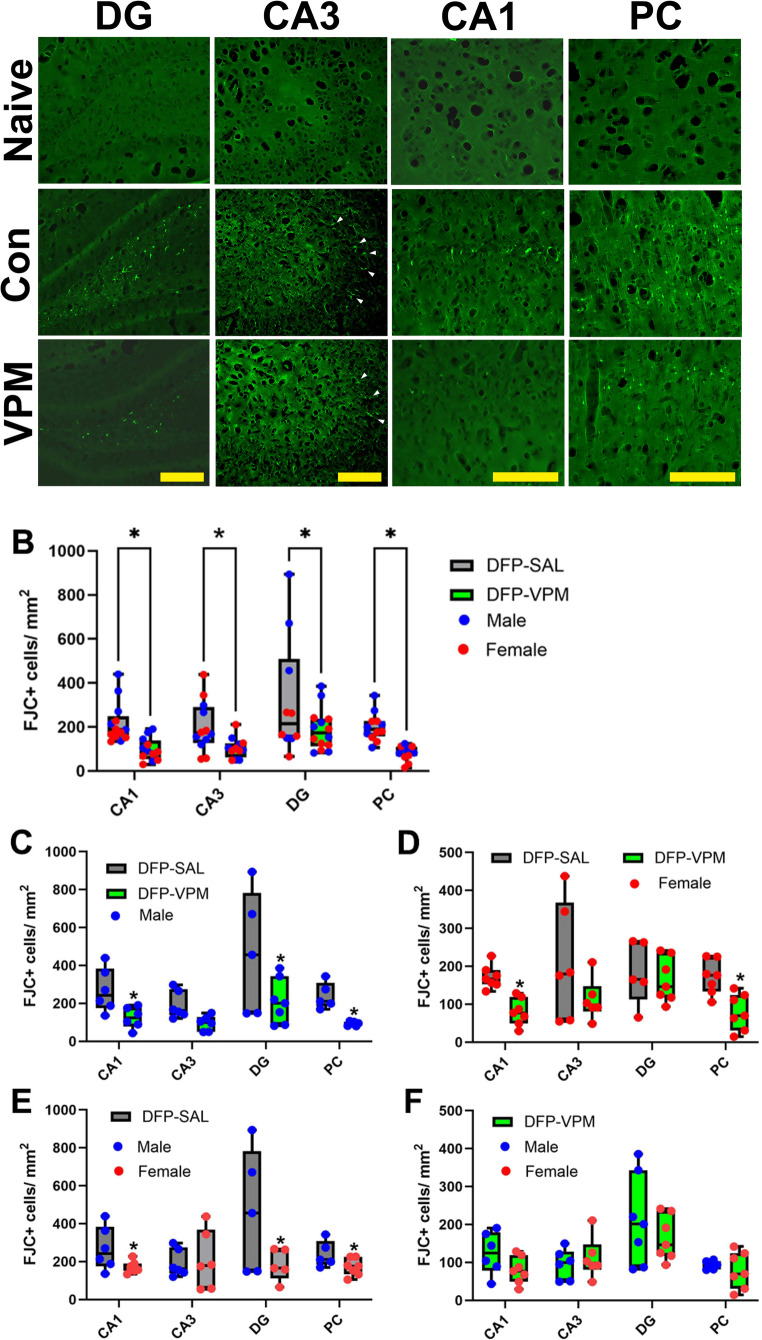
Neuroprotective effects of VPM. **(A)** Extensive FJC + staining indicative of dying neurons (depicted by white arrows in CA3) are seen in multiple brain regions including the CA1, CA3, DG, and PC from male DFP-SE rats, which is markedly reduced in VPM-treated male DFP rats (yellow bar scale 200 µM). **(B)** A significant decrease in FJC + cell counts was seen across the brain regions measured in VPM-treated DFP-SE rats compared to SAL-treated DFP-SE rats. **C-D.** Regional differences in VPM’s neuroprotective efficacy were noted between male and female rats following DFP-SE. **E.** Male rats were more sensitive to injury following DFP-SE across the CA1, DG, and PC regions. **F.** No sex differences were noted for the neuroprotective actions of VPM across all the brain regions quantified (*n* = 7–8 per group, *p <.05, two-way ANOVA, Sidak’s multiple comparison test)

Injured brain cells undergoing neurodegeneration in DFP-SE rats exhibited bright green fluorescence in FJC-stained sections (Fig. 5A). Sex-specific spatial differences in DFP-induced neuronal injury were observed (Fig. 5B-E). In the male rats, significantly more FJC + cells were found in the CA1, DG, and PC regions compared to female rats (Fig. 5E). Significantly more FJC + cells were present in the CA1, CA3, and DG regions of the hippocampus, along with the PC, in DFP rats treated with SAL compared to those treated with VPM (Fig. 5B-D). No sex differences in the neuroprotective effects of VPM were observed (Fig. 5F). No FJC + cells were found in brain sections from control rats. (Fig. 5A; *n* = 7–8 per group, *p <.05, two-way ANOVA, Sidak’s multiple comparison test).

### Stability of VPM Formulation

Stability studies based on the USP monograph for VPM injection demonstrated that our VPM injectable formulation (10 mg/mL in sterile saline) remained within 90–110% of the labeled claim at all tested time points (0, 90, and 203 days), indicating minimal degradation of VPM even after 6 months post-constitution (Table 6, *n* = 3 bottles/time point).

## Discussion

The results from our study demonstrate that i.m. VPM can achieve higher blood and brain levels and faster kinetics compared to p.o. administration in both male and female rats. Our VPM formulation had a long shelf life and underwent slight degradation even at 200 days after constitution. Adjunctive treatment regimen consisting of i.m. VPM (10 mg/kg) alongside OP-SE SOC therapy, starting 1 h after DFP-SE onset and continuing for three days, was found to be safe and did not cause significant muscle pathology either acutely or chronically. Finally, i.m. VPM therapy significantly reduced neuronal injury in multiple brain regions following DFP-SE in both male and female rats.

Since this neuroprotective adjunct treatment will initially require on-field administration, an i.m. route of administration is desirable as it provides speed for rapid intervention during a mass exposure event or battlefield-related forward care situations (Jett 2010). VPM is commonly administered p.o. in humans. We therefore first compared the PK profile of VPM in whole blood when administered p.o. and i.m. to control, no-SE rats (Table 3). Non-compartmental analyses revealed that VPM, when administered via the i.m. route, produced significantly higher drug levels and exhibited significantly greater bioavailability than when administered p.o. in both male and female rats. In contrast, time to peak VPM level (Tmax) was delayed following i.m. dosing under control conditions. However, following DFP-SE, a significantly higher Cmax was obtained compared to the Cmax following i.m. administration in control, no-SE conditions. Interestingly, the duration to Tmax obtained after i.m. administration in DFP-SE was shorter than the Tmax obtained after i.m. administration in control rats and was comparable to the values obtained in controls upon p.o. administration. Overall, these results indicated that the i.m. route provided significantly higher VPM levels, achieved stable therapeutic blood levels, and demonstrated a favorable PK profile, suggesting it could be an effective route to deliver VPM in OP-SE (Table 4; Fig. 2).

The blood-brain barrier (BBB) is a diffusion barrier that regulates substances entering the brain. In addition, P-glycoprotein (P-gp) is a transporter protein that protects the brain by limiting the penetrance of xenobiotics. These mechanisms play a role in limiting the entry and levels of drugs in the brain. In a rat model of ischemia-induced CNS injury, the level of VPM penetrance in the brain was reported to be dependent on BBB breakdown and also regulated by P-gp (Fang et al. 2013). Thus, we studied VPM brain levels following DFP-induced SE. It has been previously reported that following DFP-SE, the BBB becomes leaky and increased permeability is noticed as early as 6 h following SE and persists for up to 7 days (Bernardino et al. 2023, 2024). Furthermore, VPM is reported to bind directly to and inhibit P-gp. In our studies, under both control and DFP-SE conditions, VPM was measurable in the brain upon i.m. administration (Table 5; Fig. 3). Higher VPM brain levels were observed in both male and female rats following DFP-SE compared to the control condition. Overall, there was a trend toward greater and quicker VPM brain accumulation in males compared to females, although a statistical significance was not noted. Interestingly, VPM levels were sustained longer in female rats than male rats under both DFP-SE and control conditions. These differences in VPM brain kinetics could partially explain the sex differences seen in the neuroprotective efficacy of VPM. It may also warrant optimization of dose and frequency of administration between the two sexes. Future studies in our lab are planned to investigate these sex differences.

**Table 5:** Brain levels of VPM (ng/g) in control and DFP-SE rats

Time	control, male	DFP-SE, male	control, female	DFP-SE, female
5 min	454.1 ± 194.5	248.3 ± 86.91	121.7 ± 41.7	314.3 ± 119.3
30 min	773.5 ± 53.62	1497.0 ± 366.1	548.9 ± 142.8	778.0 ± 69.08
1 h	994.3 ± 295.3	1095.0 ± 761.6	650.9 ± 201.8	943.2 ± 382.8
2 h	532.0 ± 173.0	682.0 ± 291.6	271.6 ± 67.98	510.6 ± 244.0
4 h	171.2 ± 32.63	378.0 ± 107.7	180.9 ± 35.06	317.1 ± 45.09
6 h	54.62 ± 12.27	114.5 ± 33.74	75.82 ± 14.86	231.6 ± 97.33
12 h	-	4.65 ± 2.31	5.1 ± 2.25	32.42 ± 13.41
18 h	-	-	-	13.05 ± 6.2
24 h	-	-	-	-

Verapamil (10 mg/kg) was administered to Control and DFP-SE rats via the intramuscular (i.m.) route. Animals were sacrificed at various time points after VPM injection, and the brain was collected for analysis. Cortical VPM levels were assessed using LC-MS/MS

Significant neuronal damage has been reported following OP-SE (Deshpande et al. 2010, 2014; Gage et al. 2021; Li et al. 2011; S et al. 2025; Wu et al. 2018). Brain regions that show the most severe damage include the hippocampal regions (CA1, CA3, DG), as well as cortical regions such as the parietal cortex and the piriform cortex, along with nuclear regions in the amygdala and the thalamus. Injury to these critical structures also damages the brain circuitry and compromises the network functionality between these brain regions. In agreement with these findings, widespread neuronal damage to structures in the limbic system and cortical areas was noted following DFP-SE (Fig. 5). Sex-specific and regional differences in vulnerability to neuronal damage following DFP-SE have been previously reported. For example, one DFP-SE study reported that FJB + cells were significantly higher in the DG but not in the cortical region in adult female rats compared to adult male rats (Singh et al. 2024). Interestingly, male juvenile rats exhibited greater cell injury across all brain regions following DFP-SE compared to female juvenile rats (Gonzalez et al. 2021). In our study, FJC + cells were significantly higher in male rats in the CA1, DG, and PC regions. In contrast, female rats exhibited greater neuronal injury in the CA3 region following DFP-SE.

There are reports of neuroprotective actions of VPM in experimental models of brain injury. For example, VPM increased neuronal survival and memory in a mouse stroke model (Maniskas et al. 2016). VPM (20 mg/kg, i.p.) also prevented neuronal damage and rescued memory following severe hypoglycemia in rats (Jackson et al. 2018). VPM (10 mg/kg, i.p.) is also reported to decrease apoptosis in a rat model of ischemia/reperfusion injury (Jangholi et al. 2020). In a cellular model of Alzheimer’s Disease, destabilization of calcium homeostasis and enhanced glutamate excitotoxicity were noted, which were attenuated by VPM (Kim et al. 2000). Similarly, VPM was reported to be neuroprotective in a cellular model of dopaminergic neurotoxicity (Liu et al. 2011). VPM also rescued motor neurons by reducing endoplasmic reticulum stress in a mouse model of Amyotrophic Lateral Sclerosis (Zhang et al. 2019). Together, these findings provide a rationale for VPM as a potential therapy to attenuate OP-SE toxicities.

Our results demonstrated that VPM intervention had a significant neuroprotective action in the CA1 and PC regions for both sexes. However, VPM was protective in the DG region in only male rats. In the CA3 regions, VPM provided some neuroprotection, as evidenced by a lower number of FJC + cells in both sexes following DFP-SE; however, significance could not be achieved. It is worth noting that male rats exhibited significantly higher VPM brain levels following DFP-SE, but the drug persisted longer in females. Whether these PK-related differences contribute to the sex-related differences in neuronal injury outcomes following DFP-SE will be addressed through investigations of VPM dose and duration in a future study. It has been demonstrated that neurodegeneration following acute DFP intoxication evolves over time and neurodegeneration persists at more delayed time points. The FJC staining conducted at 96-h after the termination of DFP-SE demonstrates the acute effects of VPM intervention on degenerating neurons. Whether this action of VPM sustains over a period of time and affords long term neuroprotection will be interesting to study in future investigations.

We also investigated the pathological effects of repeated i.m. administrations of VPM by conducting muscle histology at the injection site (Deshpande et al. 2020; Patterson et al. 2008). Our studies demonstrated that the i.m. treatment regimen with VPM did not produce any overt inflammation or other pathology at the injection sites at both acute and chronic time points after the end of the injection period. No sex differences were noted in muscle pathology following i.m. VPM administrations (Fig. 4). While a few clusters of scattered infiltrates indicative of mild inflammation were noted at acute time points, the pathology scores between DFP rats treated with SAL or VPM were not different. At 3 months after the last VPM injection, the muscle pathology revealed some minimal infiltrates in some rats, while the majority had pathology scores of 0, indicating the absence of inflammation. These studies establish the safety of i.m. VPM and demonstrates that this route of administration may be an effective method to deliver VPM post-SE. Our VPM formulation had an extended shelf life and showed slight degradation even after 200 days following constitution (Table 6). The stability and ease of VPM constitution for i.m. administration will afford benefits for stockpiling VPM formulations in the event it is further optimized as an OP countermeasure.

**Table 6:** Stability of VPM formulation

Stability Time Point	% Labeled Claim
Day 0	98.21 ± 0.88
Day 90	108.54 ± 1.79
Day 203	108.65 ± 0.55

The stability of the VPM formulation was evaluated under ICH QA1 (R2) guidelines [Stability Testing of New Drug Substances and Products] and in accordance with the USP VPM injection monograph (USP29-NF24). VPM formulation (10 mg/mL in sterile saline) was stored in glass bottles at 25 °C ± 2 °C and 60 ± 5% Relative Humidity. At the respective time points, three bottles were randomly chosen, and VPM levels were assessed via HPLC methods

Our study had some limitations. First, we did not assess electrographic SE and instead behaviorally assessed SE severity on the Racine scale. We have previously shown that rats displaying a Racine score of 4 and above reliably exhibit electrographic EEG patterns of SE (Blair et al. 2024; Deshpande et al. 2010). Additionally, other studies have shown that rats from mixed-sex cohorts housed in the same room yielded reproducible SE severity in both sexes, regardless of whether they were in EEG telemetry (surgery) or non-telemetry (non-surgery) groups (Rao et al. 2022). Thus, while our study included only rats that were Racine 4 and greater (that is, high-SE responders), an EEG confirmation would have provided a more objective stratification for SE severity. Subsequently, VPM’s outcomes in such high and low SE responders could be studied for a better understanding of therapeutic efficacy. Second, we did not quantify neuroinflammatory changes in this study. While our data showed strong neuroprotective actions of VPM following DFP-SE, neuroprotection is a complex process involving many mechanisms beyond neuroinflammation. Neuroinflammatory changes are reported in SE and DFP-SE while VPM is reported to exert anti-inflammatory actions in laboratory studies. Whether the neuroprotective effects noted here are achieved through an anti-inflammatory response will be a critical piece of information to understand VPM’s protective actions in DFP-SE fully. While, the absence of inflammatory biomarkers here limits the interpretation of VPM’s neuroprotective action, it does not invalidate it entirely. Indeed, the goal of this study was to validate the neuroprotective efficacy of a VPM formulation that could be safely administered i.m. after a reasonable delay following DFP-SE. Studies are underway in our laboratory to investigate the anti-inflammatory effects of VPM and its underlying molecular mechanism. Third, while our studies showed favorable PK properties following a single dose of i.m. VPM, expanded analysis in an even larger sample size and following multiple VPM doses could offer additional details on PK profile of i.m. VPM following DFP-SE. Fourth, we utilized VPM at a single dose (10 mg/kg, i.m.). As discussed previously, this dose was selected as being closer to human-equivalent concentrations. Whether a higher VPM dose or a longer duration of VPM therapy would improve SE-induced neuropathology outcomes will be systematically evaluated in future studies. The clinical translation of these findings should consider the limitations of the animal model. As with pre-clinical animal studies, animal models don’t fully reflect the complexity of human diseases. Metabolic and physiologic differences can affect how a toxin exacerbates injury and how a protective therapy in animals may lack efficacy in humans. The OP toxicity research has an additional roadblock since clinical trials for countermeasures for nerve agents would be unethical. Thus, repurposing existing FDA-approved drugs and demonstrating efficacy in animal studies are critical steps in nerve agent countermeasures research. Thus, the effects of an optimized VPM therapy on long-term neuropathological, behavioral, and seizure outcomes will provide clear translational potential of this proposed VPM-based therapy against OP-induced toxicity. In conclusion, the data from this study demonstrate the potential for repurposing VPM as an effective adjunct to existing SOC pharmacological countermeasure therapy for extending neuroprotective efficacy and improving long-term neurological outcomes after OP intoxication.

## Data Availability

No datasets were generated or analysed during the current study.

## Abbreviations

2-PAM: Pralidoxime chloride
AChE: Acetylcholinesterase
b.i.d.: bis in die (English- twice-daily)
DFP: Diisopropyl Fluorophosphate
EEG: Electroencephalogram
FDA: Food and Drug Administration
FJC: Fluoro-Jade C
i.m.: intramuscular route
OP: Organophosphate
p.o.: oral route
PK: Pharmacokinetic
s.c.: subcutaneous route
SAL: Saline
SE: Status Epilepticus
SOC: Standard-of-Care
VPM: Verapamil
